# The statistical interpretation of pilot trials: should significance thresholds be reconsidered?

**DOI:** 10.1186/1471-2288-14-41

**Published:** 2014-03-20

**Authors:** Ellen C Lee, Amy L Whitehead, Richard M Jacques, Steven A Julious

**Affiliations:** 1 Medical Statistics Group, School of Health and Related Research (ScHARR), University of Sheffield, 30 Regent Street, Sheffield S1 4DA, UK

**Keywords:** Pilot trial, Power, Type I error, Confidence interval, Significance, Bayesian methods

## Abstract

**Background:**

In an evaluation of a new health technology, a pilot trial may be undertaken prior to a trial that makes a definitive assessment of benefit. The objective of pilot studies is to provide sufficient evidence that a larger definitive trial can be undertaken and, at times, to provide a preliminary assessment of benefit.

**Methods:**

We describe significance thresholds, confidence intervals and surrogate markers in the context of pilot studies and how Bayesian methods can be used in pilot trials. We use a worked example to illustrate the issues raised.

**Results:**

We show how significance levels other than the traditional 5% should be considered to provide preliminary evidence for efficacy and how estimation and confidence intervals should be the focus to provide an estimated range of possible treatment effects. We also illustrate how Bayesian methods could also assist in the early assessment of a health technology.

**Conclusions:**

We recommend that in pilot trials the focus should be on descriptive statistics and estimation, using confidence intervals, rather than formal hypothesis testing and that confidence intervals other than 95% confidence intervals, such as 85% or 75%, be used for the estimation. The confidence interval should then be interpreted with regards to the minimum clinically important difference. We also recommend that Bayesian methods be used to assist in the interpretation of pilot trials. Surrogate endpoints can also be used in pilot trials but they must reliably predict the overall effect on the clinical outcome.

## Background

In an evaluation of a new health technology, a pilot trial may be undertaken prior to a definitive trial that makes a definitive assessment of benefit. The main objective of a pilot trial is to provide sufficient assurance to enable a larger definitive trial to be undertaken. For example, they may assess aspects such as recruitment rates or whether the technologies can be implemented.

Pilot studies are more about learning than confirming: they are not designed to formally assess evidence of benefit. As such, for clinical endpoints, rather than formal hypothesis testing to prove definitively there is a response, it is usually more informative to provide an estimate of the range of possible responses [1,2]. This estimation may not be around the primary endpoint for the definitive study but could be on a surrogate or an early assessment of an endpoint which may be assessed at a later time point in the definitive study [3].

In this paper we present and discuss approaches towards significance thresholds and confidence interval levels in pilot studies. The methods are divided into three main sections. In the first, we provide alternatives to hypothesis testing using the conventional 5% significance level. We then discuss the use of surrogate outcomes in pilot studies. Finally, a Bayesian approach to significant thresholds is introduced. Throughout the paper we use a worked example to provide illustration to the methods discussed.

## Methods and results

### Significance and confidence levels

Pilot studies are not formally powered to assess effect. However, it may be of interest to calculate confidence intervals to describe the range of effects, even if this is not a conventional 95% confidence interval. In this section we give a rational for confidence interval estimation and “hypothesis testing” in pilot studies.

### Significance levels and power calculations

Pilot studies are usually underpowered to achieve statistical significance at the commonly used 5% level. Despite recommendations that formal significance levels are not provided for pilot studies, [4,5] many still quote and interpret P-values. In a survey of pilot studies published in 2007–8, Arain et al. [6] found that 81% (21/26) of pilot studies performed hypothesis tests in order to comment on the statistical significance of results. If the primary purpose of a pilot study is to provide preliminary evidence of the efficacy of an intervention, then the significance level can be increased for hypothesis testing [7]. Stallard [8] recommends that the design for a phase II trial is based on a one sided Type I error rate of α = 0.2. Whilst Schoenfeld [9] proposed a higher type I error rate for preliminary testing in pilot trials; up to a (one sided) α = 0.25. In studies other than drug trials, setting and personnel may not be representative of a future main trial: A pilot trial might see a greater treatment difference due to protocol adherence and enthusiasm in the pilot centre, which might not be replicated in a multi-centre trial. Nevertheless, the pilot may still be underpowered for a traditional 5% significance threshold.

It should be noted that in the context of a pilot study a Type I error would have a different impact. For a definitive study, a Type I error would mean therapies or health technologies falsely being concluded as beneficial. As such, in this context they would be referred to as societies risk – such that the wish is to have a Type I error as low as possible. For a pilot study the impact of a Type I error is that a definitive study may falsely be undertaken. Although there is a consequence for patients in the trial – being randomised to therapies when there is equipoise – the impact of this false positive error could be in the main on the sponsor or funder i.e. sponsors spend more money and resources on the ‘wrong’ study that will not result in a true effect/benefit from the new technology.

The aim of a pilot study, therefore, is to inform both the decision whether to conduct a confirmatory study and the design of the larger confirmatory trial. Any interpreted P-values in a pilot study should be with a disclaimer that the study is not adequately powered [10,11]; and while *post hoc* power calculations are possible [11] they are generally not advisable [12]. Instead, estimation and confidence intervals should be used to infer the size and direction of treatment effect.

### Confidence intervals

It is recommended in pilot trials that the focus is on descriptive statistics and estimation rather than formal hypothesis testing [4]. A confidence interval for the treatment effect will inform the decision, amongst other factors, whether or not to perform a confirmatory trial. The confidence interval should be interpreted with regards to the minimum clinically important difference (MCID) [12]; this is the difference between treatment groups that is considered to be clinically meaningful, specified *a priori*. If a confidence interval for the treatment difference crosses zero and the MCID, then the results of the pilot study could be considered to be equivocal. There could be no difference between treatments, or there could be a difference larger than the MCID; the results would not preclude either possibility. This approach is superior to formal hypothesis testing as there is insufficient power to test hypotheses, and its focus on the MCID will help inform the main confirmatory trial. Interpreting confidence intervals this way also helps investigators visualise the evidence of effect from the pilot trial.

It is common to report the 95% confidence interval which corresponds to a 5% significance level. In a pilot study, without adequate power, we can consider investigating confidence intervals of different widths to help inform our decision making, these can then be displayed alongside each other to illustrate the strength of preliminary evidence. We suggest setting minimum prior requirement; that the mean treatment difference is above zero, and that a CI of a certain length includes (or is above) the MCID.

### Worked example

The Leg Ulcer Study was a randomised controlled trial designed to investigate the relative cost effectiveness of community leg ulcer clinics that use four layer compression bandaging versus usual care provided by district nurses [13,14]. In the trial 233 patients with venous leg ulcers were allocated at random to the intervention (120) or control (113) group. The SF-36 questionnaire was completed at baseline, three and twelve months post randomisation. For this example we investigate the SF-36 General Health (GH) dimension score. The GH dimension is scored on a 0 (poor) to 100 (good health) scale.

We assume that 3 month data for the first 40 patients is the pilot study data. There were 31 individuals with complete 3 month SF-36 GH dimension data (17 in treatment group and 14 in control group).

Note missing data on 22.5% (9/40) patients is quite high and may be considered unacceptable for a main study. In actuality for this trial there was just 14% (29/230) of missing data for the SF-36 data [15]. For our data we may well have observed a randomly high number. If this was a true pilot study then a missing data rate of 22.5% may need some investigation. There are statistical methods for accounting for missing data [16]. However, the only solution to missing data is not to have any. After a pilot study, measures to ensure complete data would need to be investigated to bring the level of missing data to an acceptable level.

We take the minimum clinically important difference to be a 5 point difference in SF-36 GH dimension scores at 3 months post-randomisation; we assume a standard deviation of 20 points. Without seeing the actual trial results, with 40 individuals, there would be 20% power to detect a 5 point or more difference between the groups if it truly existed which is clearly underpowered by conventional standards. Thus, for such a trial it would be more appropriate to estimate possible effects rather than have formal hypothesis tests.

Table 1 displays the results comparing the mean SF-36 GH dimension scores between the home (control) and clinic (intervention) group. The mean difference was found to be 12.8, which is statistically significant at the 10% but not 5% level; there is some evidence of a difference in SF-36 GH dimension between groups. If the significance level was set to 10%, there would be sufficient preliminary evidence of a treatment difference and this would lead onto a full-scale study.

**Table 1:** Results from the pilot study comparing 3-month SF-36 GH dimension scores

**Mean SF-36 GH dimension score**			
**Clinic (n = 17)**	**Home (n = 14)**	**Difference (95% CI)**	**P-value**
68.0 (sd = 17.6)	55.1 (sd = 19.8)	12.8 (−0.8 to 26.6)	0.065

The leg ulcer randomised controlled trial reported in 1998 obtained appropriate ethics committee approvals [14]. The use of the data from this trial for the work presented in this paper has been approved by School of Health and Related Research (University of Sheffield) ethics as secondary analysis of anonymised data.

**Figure 1:**
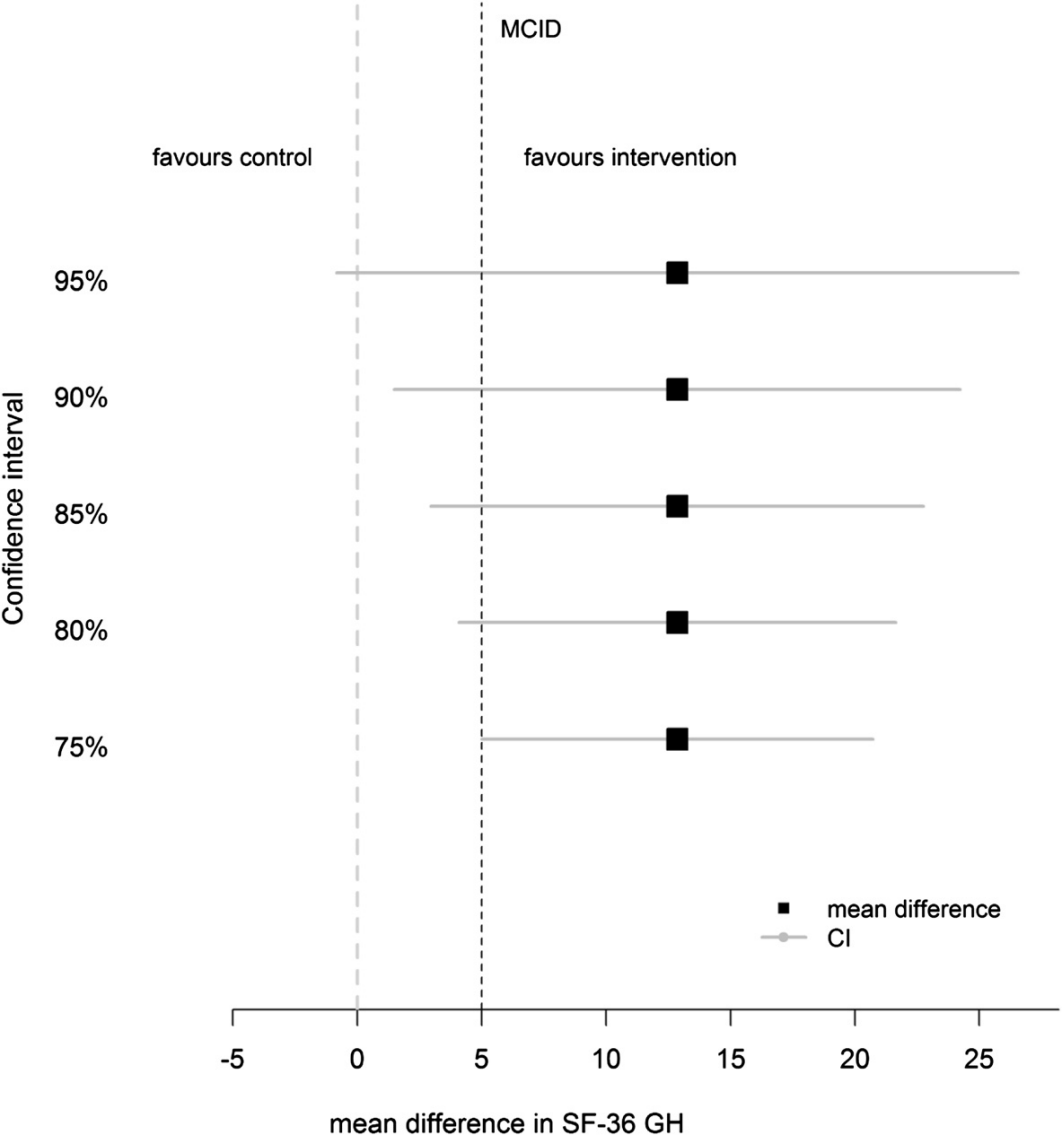
Mean difference in SF-36 GH dimension scores between treatment and control with confidence intervals (based on n = 31 patients).

Figure 1 shows a range of confidence intervals for the mean difference in SF-36 GH scores between the treatment groups. The 95% CI crosses both 0 and the MCID, this gives inconclusive evidence. The 80% and 90% confidence intervals both exclude 0 and cross the MCID, at these levels there is evidence of a treatment difference which is potentially clinically important. A confidence interval of 75% and smaller would be wholly above or equal to the MCID, suggesting at this level that there is a clinically meaningful difference in SF-36 General Health between the groups.

### Outcomes

The NIHR Evaluation, Trials and Studies Coordinating Centre (NETSCC) describes a pilot study as a smaller version of the main trial, designed to test whether components of the main study can all work together as well as a preliminary assessment of clinical efficacy. This screening function of pilot studies requires a preliminary evaluation of treatments. Therefore, using the definitive clinical endpoint during a pilot trial may not always be viable. There may be times when measuring the clinical endpoint is not efficient [17]. For example, if the clinical endpoint is the five year survival rate, then an assessment of disease progression or tumour shrinkage may be assessed in the pilot. Such endpoints would be used as surrogates for the definitive endpoint. We will now discuss surrogates in more detail [18].

### Surrogate endpoints

In the situations described above an investigator may consider using an endpoint other than the clinical endpoint; a surrogate endpoint. ICH E9 [19] defines a surrogate endpoint as

‘A variable that provides an indirect measurement of effect in situations where direct measurement of clinical effect is not feasible or practical’.

Using a surrogate endpoint can reduce the required sample size or the duration of the trial compared to using the clinical endpoint. This leads to cost reductions which may be crucial for trial feasibility [18]. For an endpoint to be considered a surrogate the relationship between it and the clinical outcome must be biologically plausible. In addition, the surrogate must have demonstrable prognostic value for the clinical outcome and there must be evidence from clinical trials that treatment effects on the surrogate outcome correspond to treatments effects on the clinical outcome [19].

### The risks involved when using surrogate endpoints

When an aim of a pilot study is to estimate design parameters, using a surrogate endpoint may mean we do not get precise estimates. For example, designing the study based on the surrogate may mean having sub optimal information to estimate the variance of the clinical endpoint or an assessment at an earlier time point. This may mean we do not get an accurate estimate of attrition rates.

A surrogate endpoint must reliably predict the overall effect on the clinical outcome [20]. Otherwise it would be possible to wrongly reject effective treatments or take ineffective treatments through to further testing. If a surrogate does predict clinical benefit it could mean treatment benefits can be brought to patients earlier than if clinical outcomes were used and possibly at a lower cost [21].

### Worked example revisited

Using the same data set as in the previous example we now look at the 12 month SF-36 general health (GH) dimension data for the main trial. There were 233 people in the study in total, 155 with complete SF-36 GH dimension data and 78 observations were recorded as missing. From the 155 observed outcomes 80 were in the clinic group and 75 were in the home or control group – note we had 23% attrition at 3 months compared to 31% at 12 months. Such considerations may be important when trying to design a definitive trial.

Table 2 presents the results from comparing the mean SF-36 GH dimension scores between home and clinic groups. The mean difference was 3.33 which is not significant at the 5% level. The original presentation of these results in 1998 stated that they observed a general deterioration of health status over time, with no difference between the two groups [14].

**Table 2:** Results from main trial comparing 12-month GH dimension scores

**Clinic (n = 80)**	**Home (n = 75)**	**Difference (95% CI)**	**P-value**
56.0 (sd = 22.8)	52.7 (sd = 23.9)	3.3 (−4.1 to 10.8)	0.377

Mean SF-36 GH dimension score.

In the previous worked example we envisaged that the pilot trial had 40 patients and measured the 3-month GH dimension score. Using a significance level of 10% we would have proceeded to the main trial. The 3-month GH dimension score is now considered as a surrogate endpoint to the clinical outcome of 12-month GH dimension score. If we used a significance level of 5% to assess the clinical outcome, the difference between the groups is not statistically significant. Using the 3-month endpoint in the pilot study and a lower significance level would cause us to proceed to the main trial after the pilot study only to observe no significant difference between the two groups in the main study. It could be a Type I error which would lead us to the main study or it could be due to the treatment having no long term efficacy – for example the intervention may have a short term benefit which does not last for 12 months. The ‘large’ effect of 12.8 points in the first 40 patients at 3 months has not been replicated at 12 months in the full study.

### Bayesian methods

The Bayesian framework offers an alternative approach to the Frequentist significance levels and confidence intervals discussed in the previous section. It allows prior beliefs about the intervention to be combined with the observed data to form posterior responses about the outcome of interest. These posterior responses can then be used to inform decisions about whether a larger definitive trial should be undertaken. One approach to making a decision about the intervention is to use a pre-specified Go/No-Go criteria.

### Go/No-Go criteria

Julious et al. [22] define a Go/No-Go decision as a hurdle in a clinical development path to necessitate further progression or otherwise of a health technology. These hurdles can be set low or high depending on the stage of development of the intervention.

At the planning stage of a pilot study there are a number of decisions that need to be made about how Go/No-Go criteria are defined. The first concerns the metric that is going to measure success or failure. Julious and Swank [23] suggest a method of calculating a probability of success for different development plans based on decision trees and Bayes’ Theorem. They take into account the study team’s confidence (expressed as a probability) that the intervention will meet the safety and efficacy targets for success, and then calculate the probability that each part of the clinical assessment will correctly indicate that the health technology works or does not work.

Chuang-Stein et al. [24] suggest that a good metric is the probability that there will be a successful confirmatory trial outcome. This is also called assurance by O’Hagan et al. [25] or average power by Chuang-Stein [26] and is used in Bayesian sample size calculations for confirmatory trials. The method that we describe here in detail uses prior beliefs and the data collected from the pilot study to calculate the probability of detecting a clinically meaningful difference. This method has previously been described by Julious et al. [22] for binary and Normal outcomes, and Parmar et al. [27] for survival outcomes.

The second decision concerns the cut-off or level of the criteria. For example, do we want to be 70% or 80% sure that a confirmatory trial will show a minimum clinically meaningful difference? With a pilot study, criteria could be set to minimise the probability of a false positive, (i.e. minimising the probability of progressing an intervention that will fail in a confirmatory trial) but if the goal is set too high then this will increase the probability of a false negative (i.e. stopping an intervention that works from going to a confirmatory trial) [22]. Other factors may also influence the choice of criteria, for example, the sponsor of a drug trial may be more willing to accept an incorrect go decision rather than an incorrect no-go decision if the new treatment is the first in class rather than one of several drugs in class [24].

### Prior distributions

As with all Bayesian methods, prior distributions have to be specified for the parameters that we are interested in making inference about and this leads to the question of how these distributions are defined. The simplest approach is to use a non-informative prior. In this case the results will be similar to the Frequentist analysis because all of the information is coming from the observed response. Alternatively, a prior can be elicited based on expert knowledge of the intervention. This may, for example, be based on the synthesis of evidence from previous studies of the same or similar interventions as suggested by Chuang-Stein et al. [24]. Other elicitation techniques including the elicitation from multiple experts are discussed in Spiegelhalter et al. [28].

With a large sample size for the pilot study the posterior distribution will be robust to changes in the prior [29]. However, sample sizes in pilot studies are typically small - in a literature survey by Arain et al. [6] the median number of participants was 76 - and therefore an informative prior distribution may have a large influence on the posterior distribution. We illustrate in our example that caution should be taken when specifying a prior distribution for a pilot study, as different priors may lead to different interpretations of the results.

### Probability of detecting a clinically meaningful difference

We now outline one possible method for calculating the probability of detecting a clinically meaningful difference for data that are anticipated to take a Normal form. In the context of a Go/No-Go criteria we need to determine the probability of observing a difference, d_i_, or greater given that d_pilot_ has already been observed, i.e. prob(θ > d_i_ | d_pilot_) where θ is the mean difference.

For Normal data of the form X_1_,X_2_,…,X_n_ ~ N(θ, σ^2^) we wish to make inference about θ for given σ^2^. In this case the Normal family is conjugate and we have the following prior θ ~ N(μ_prior_, σ_prior_^2^). Note that other distributions may be used for the prior. The Bayesian updating rules can then be defined as follows.

Prior values for the mean difference and population standard deviation are defined as d_prior_ and s_prior_ respectively. The observed mean difference and population standard deviation from the pilot data are defined as d_pilot_ and s_pilot_ respectively. Hence $S_{1}\sqrt{\left(r+1\right)/\mathrm{rn}}$ is an estimate of the standard deviation around the mean where r is the allocation ratio between groups and n is the number of individuals per arm.

The posterior distribution is calculated through a weighted sum of the prior and observed responses. The posterior estimate of the mean difference, d_post_, is defined as

$$d_{\mathrm{post}}=s_{\mathrm{post}}^{2}\left(\frac{d_{\mathrm{prior}}}{s_{\mathrm{prior}}^{2}}+\frac{d_{\mathrm{pilot}}\mathrm{rn}}{s_{\mathrm{pilot}}^{2}\left(r+1\right)}\right)$$

and the posterior estimate of the variance around the mean, $s_{\mathrm{post}}^{2}$, is defined as

$$S_{\mathrm{post}}^{2}={\left(\frac{\mathrm{rn}}{s_{\mathrm{pilot}}^{2}\left(r+1\right)}+\frac{1}{s_{\mathrm{prior}}^{2}}\right)}^{-1}.$$

From these posterior values a density distribution for prob(θ > d_i_ | d_pilot_) can be defined so that the probability of observing a difference, d_i_, or greater, for a given d_post_ would be

$$\mathrm{prob}\left(\theta >d_{i}\left|d_{\mathrm{pilot}}\right)\right)=\Phi \left(\frac{d_{i}-d_{\mathrm{post}}}{s_{\mathrm{post}}}\right).$$

### Worked example revisited with bayesian approach

Using the same leg ulcer data as described previously, we demonstrate how to calculate the probability that the mean difference in SF-36 GH dimension scores at 3 months post randomisation is greater than the minimum clinically important difference of five points. This question may also be stated in terms of a ‘Go’ criteria, for example:

Are we at least 75% sure of having a mean difference in SF-36 GH dimension that is greater than the minimum clinically meaningful difference of five points at 3 months post randomisation.

For the expository purpose of this exercise we will consider the following three Normally distributed priors:

•Non-informative

•Pessimistic prior, with a mean difference of 4 and 90% certainty that the mean difference is within −1 and 9.

•Optimistic prior, with a mean difference of 7 and 90% certainty that the mean difference is within 4 and 10.

Table 3 displays the posterior mean, posterior standard deviation, and the probability that the mean difference in SF-36 GH dimension score is greater than the minimum clinically meaningful difference of 5 points for our examples of a non-informative, pessimistic and optimistic prior distribution. When using both the non-informative and the optimistic prior the probability of achieving a clinically meaningful difference is greater than our pre-set threshold of 75%.

**Table 3:** Posterior means, standard deviations and the probability of observing a clinically meaningful effect size of greater than 5 for non-informative, pessimistic and optimistic priors

**Prior**	**Posterior mean**	**Posterior SD**	**P(>5)**
Non-Informative	12.9	6.7	0.88
Pessimistic	5.5	2.8	0.58
Optimistic	7.4	1.8	0.91

**Figure 2:**
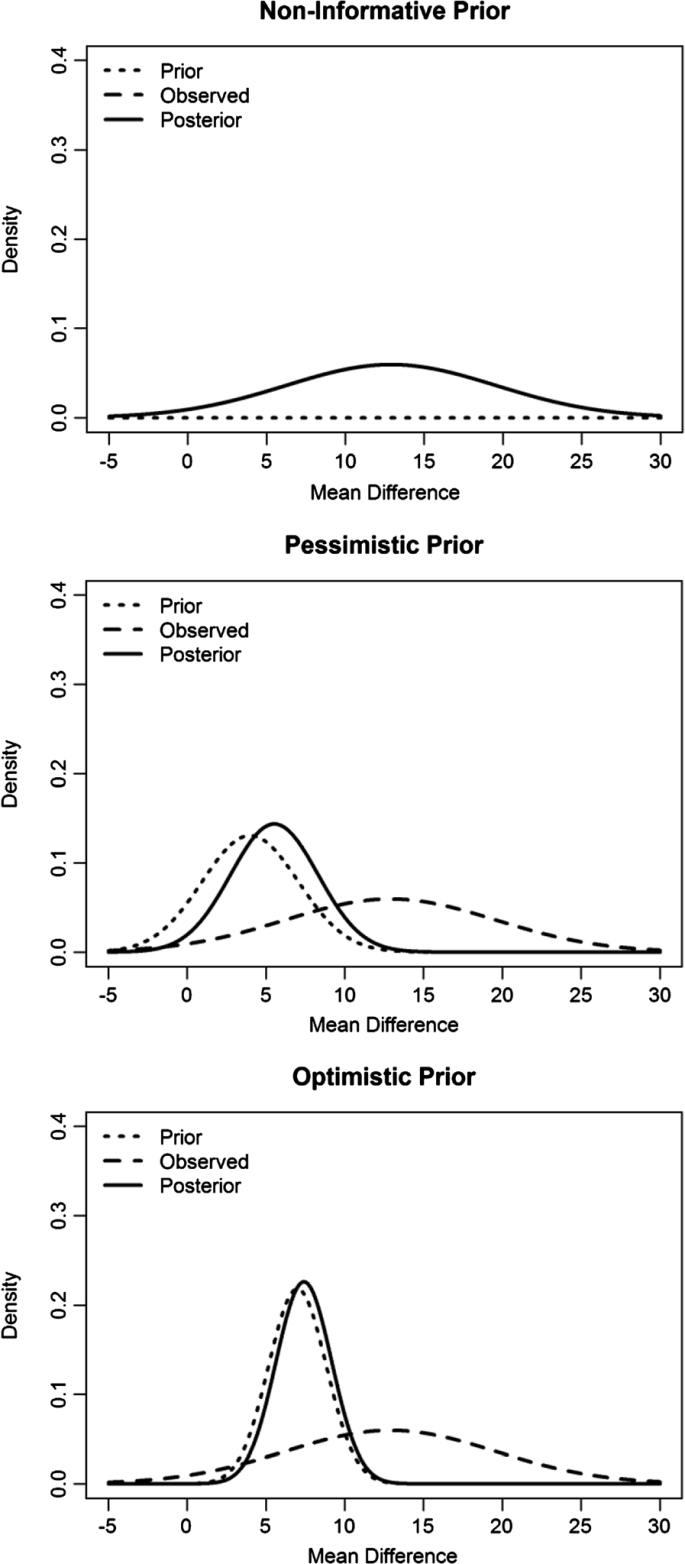
Prior, observed and posterior distributions for non-informative, pessimistic and optimistic priors.

Figure 2 shows the prior, observed, and posterior distributions for each of our three examples. The non-informative prior has no influence on the posterior distribution and the 95% credibility interval for the posterior mean difference is the same as 95% confidence interval found previously (−0.8 to 26.6). In the case of the pessimistic and optimistic priors the posterior distribution is heavily influenced by the choice of prior because the observed data has such a small sample size. This emphasises that caution is required when specifying a prior distribution for pilot studies.

It could be argued that a Bayesian approach is appealing as it formally accounts for any related work (and/or of beliefs held by investigators) by setting priors before the start of a study [22]. Once the trial has been completed, the observed data are combined with the priors to form a posterior distribution for the treatment response. The interpretation is then through a measure that is more easily understood – in our example what is the probability that the response is greater than 5.

## Discussion

This paper has demonstrated a variety of approaches towards significance thresholds in pilot studies. When undertaking a pilot investigation, it was shown how significance levels other than the “traditional” 5% should be considered to provide preliminary evidence for efficacy. It was highlighted how estimation and confidence intervals should be focused on in order to provide an estimated range of possible treatment effects.

Interpreting confidence intervals with respect to the minimum clinically important difference should be considered. Investigating several confidence intervals of different widths and displaying them as in Figure 1 can aid decision making and is a helpful way of displaying evidence in pilot studies. Minimum prior requirements can be set and used in addition to the graphical display to help illustrate the strength of preliminary evidence. However, caution must be taken when using a surrogate outcome in pilot studies as it must reliably predict the clinical endpoint.

Bayesian methods could also assist in the early assessment of a health technology. Pilot data can be combined with prior beliefs in order to calculate the probability that there will be a successful confirmatory trial outcome. This can be framed into a Go/No-Go hurdle such as; *are we at least 75% sure of having a mean difference larger than the minimum clinically meaningful difference*. We demonstrated how care must be taken when choosing a prior distribution; the posterior distribution can be heavily influenced by the choice of prior as pilot data usually has a small sample size.

## Conclusions

We recommend that in pilot trials the focus should be on descriptive statistics and estimation, using confidence intervals, rather than formal hypothesis testing. We further recommend that confidence intervals in addition to 95% confidence intervals, such as 85% or 75%, be used for the estimation. The confidence interval should then be interpreted with regards to the minimum clinically important difference and we suggest setting minimum prior requirements. Although Bayesian methods could assist in the interpretation of pilot trials, we recommend that they are used with caution due to small sample sizes.

## Abbreviations

GH: General Health; MCID: Minimum Clinically Important Difference; NETSCC: National Institute for Health Research Evaluation, Trials and Studies Coordinating Centre.

## Competing interests

The authors declare that they have no competing interests.

## Authors’ contributions

All authors contributed equally to the work in this paper. All authors read and approved the final manuscript.

## Pre-publication history

The pre-publication history for this paper can be accessed here:

http://www.biomedcentral.com/1471-2288/14/41/prepub
